# Attenuated Increase in Maximal Force of Rat Medial Gastrocnemius Muscle after Concurrent Peak Power and Endurance Training

**DOI:** 10.1155/2013/935671

**Published:** 2013-01-27

**Authors:** Regula Furrer, Richard T. Jaspers, Hein L. Baggerman, Nathalie Bravenboer, Paul Lips, Arnold de Haan

**Affiliations:** ^1^MOVE Research Institute Amsterdam, Faculty of Human Movement Sciences, VU University Amsterdam, Van der Boechorststraat 9, 1081 BT Amsterdam, The Netherlands; ^2^Department of Clinical Chemistry, MOVE Research Institute Amsterdam, VU University Medical Center Amsterdam, 1007 MB Amsterdam, The Netherlands; ^3^Department of Endocrinology, MOVE Research Institute Amsterdam, VU University Medical Center Amsterdam, 1007 MB Amsterdam, The Netherlands; ^4^Institute for Biomedical Research into Human Movement and Health, Manchester Metropolitan University, Manchester M5 1GD, UK

## Abstract

Improvement of muscle peak power and oxidative capacity are generally presumed to be mutually exclusive. However, this may not be valid by using fibre type-specific recruitment. Since rat medial gastrocnemius muscle (GM) is composed of high and low oxidative compartments which are recruited task specifically, we hypothesised that the adaptive responses to peak power training were unaffected by additional endurance training. Thirty rats were subjected to either no training (control), peak power training (PT), or both peak power and endurance training (PET), which was performed on a treadmill 5 days per week for 6 weeks. Maximal running velocity increased 13.5% throughout the training and was similar in both training groups. Only after PT, GM maximal force was 10% higher than that of the control group. In the low oxidative compartment, mRNA levels of myostatin and MuRF-1 were higher after PT as compared to those of control and PET groups, respectively. Phospho-S6 ribosomal protein levels remained unchanged, suggesting that the elevated myostatin levels after PT did not inhibit mTOR signalling. In conclusion, even by using task-specific recruitment of the compartmentalized rat GM, additional endurance training interfered with the adaptive response of peak power training and attenuated the increase in maximal force after power training.

## 1. Introduction

Optimal physical performance, during for instance cycling and rowing, requires both high peak power and maximal oxidative capacity of skeletal muscles. Hickson (1980) was the first to study the effects of concurrent strength and endurance training (further referred to as concurrent training) aiming at increasing both maximal force and endurance [1]. During concurrent training, the gain in maximal muscle force (*F*_max⁡_) was shown to be attenuated, whereas no negative effect on the maximal oxygen uptake (*V*O_2_max⁡) was observed [1]. Since the study of Hickson (1980), the effects of concurrent training on *F*_max⁡_ have been intensively studied in humans, supporting as well as contradicting the findings of Hickson [1–7]. Recently, several interactions in the molecular regulation of the synthesis of contractile proteins and biosynthesis of mitochondria have been uncovered, suggesting that muscle fibres seem to be limited in increasing size (and therefore maximal force) and oxidative capacity simultaneously [8–11]. Although the two training modalities interfere with one another at the cellular level, the entire muscle may be able to adapt to both types of training without a negative effect on *F*_max⁡_ in response to the additional endurance training [5, 7]. However, the underlying mechanisms are still unclear and the question remains as to how these two training modalities can best be combined to improve peak power and endurance simultaneously.

A possible mechanism may be the use of specific recruitment of muscle fibres during different tasks. For instance, motor units consisting of slow high oxidative muscle fibres may be recruited during endurance training and increase the oxidative capacity, whereas motor units consisting of fast and large muscle fibres may primarily be used during peak power training and increase in size and therefore contribute to the increase in *F*_max⁡_. Such task-specific recruitment and adaptation of motor units may be studied in rat medial gastrocnemius muscle (GM), as this muscle is composed of high and low oxidative compartments which are recruited tasks specifically [12]. During low intensity activities, the high oxidative compartment is active, whereas only during high intensity activities, such as sprinting, also the low oxidative compartment is recruited [12, 13]. The aim of the current study was to investigate whether by using task-specific recruitment of high and low oxidative muscle fibres the effects of peak power training on this muscle was attenuated when combined with endurance training. We hypothesised that in compartmentalized rat GM, increases in maximal muscle force and muscle fibre size were not attenuated by additional endurance training compared to peak power training alone. In addition, in the low oxidative compartment, after both peak power training and concurrent training, we expected a similar increase in mRNA expression levels of genes inducing hypertrophy as well as reduction in mRNA levels of genes stimulating protein degradation.

## 2. Materials and Methods

### 2.1. Animal Care and Experimental Design

The experiment was approved by the Animal Experiments Committee of the VU University Amsterdam and animals were kept according to the guidelines of animal care. The study was conducted with 30 female Wistar rats at the age of 10 weeks; food and water were provided *ad libitum*. The rats were subjected to either peak power training (PT; *n* = 10), combined peak power training with additional endurance training (PET; *n* = 10), or remained untrained (control; *n* = 10). In order to train the rats during their active period of the day, the 12-h light: 12-h dark cycle was reversed. During a three week acclimatization period, rats of the training groups were familiarized with running and/or sprinting on a motor driven treadmill. Following this, the 6-week training period started, during which the rats of the PT and PET group trained 5 days per week. For studying effects on bone mineralization (not reported here) 9 and 2 days before sacrificing, a very small dose of tetracycline (25 mg/kg) was injected intraperitoneally (i.p.). To standardize for the gene expression and to exclude the acute effects of one training bout, the last training was performed approximately 22 hours before sacrificing (peak power training for the PT group and endurance training for the PET group).

### 2.2. Training Protocols

*Peak power training* consisted of 10 sprints of 15 seconds at near maximal velocity with 3 minutes of rest between the sprints. During these sprints, the rats were in gallop resembling explosive repetitive jumps of the hind limbs. Once in gallop, the speed was increased up to a velocity at which the rat could not stay in the front of the treadmill.The slope of the treadmill was progressively increased throughout the 6 weeks of training, starting at 10% and rising to 40%. To fulfil the criteria of a successful training, the rats had to attain near maximal velocities while they were in gallop. When such velocities (due to poor coordination or lack of motivation) were not attained, the training was not counted as successfully completed. For inclusion in this study, every week at least four training sessions had to be successfully completed. All rats (PT and the PET groups) were subjected to the peak power training in the morning to ensure that they had recovered from the last training session the day before. The PET group performed additional endurance training on the same day in the afternoon (8 hours later). The *endurance training* consisted of training sessions in which the duration was increased progressively from 10 minutes up to 45 minutes after 6 weeks and the inclination and velocity were increased from 0% to 10% and from 16 m/min to 26 m/min (trotting), respectively. Rats that did not sustain the training duration at the given inclination and speed during at least three training sessions, were excluded from this study.

### 2.3. Functional Performance Test

Functional performance was defined as the maximal running velocity attained at a 40% inclination. The PT and PET groups performed a maximal running velocity test at 40% inclination consisting of several sprint of 15 seconds every two weeks. The velocity started at 36 m/min and was increased with each sprint by steps of approximately 5 m/min up to their maximal velocity (with 3 minutes rest between the sprints). Since performing maximal sprints was a coordinative difficult task, requiring lots of practice, the control group did not perform the maximal running velocity tests to prevent measuring only their skills, which would result in an underestimation of their real maximal running velocity. For the same reason, the first functional performance test was performed after 2 weeks.

### 2.4. Experimental Protocol

To measure the contractile muscle force characteristics *in situ*, which took place one day after the last training, rats were anesthetized by an initial dose of 1.2 mL/100 g body weight urethane (i.p.) [14]. Within the following hour, they received 2-3 injections (i.p.) of 0.5 mL urethane (until nociceptive reflexes disappeared). The body temperature of the rat was maintained at 37°C. The right medial gastrocnemius muscle (GM), including the Achilles tendon with a piece of the calcaneus, was separated from the surrounding tissue leaving the proximal origin, the blood supply, and nerves intact. The sciatic nerve was exposed carefully and severed approximately 2 cm proximal of the GM. Nerve branches from the sciatic nerve, innervating other muscles than the GM, were also severed. The right hind limb was fixed using a metal clamp at the femur, the Achilles tendon was attached to a force transducer, and the nerve was placed over electrodes. During the experiment, the muscle and its surrounding were kept moist at physiological temperature (34-35°C). The experimental setup was similar as described previously [12]. The contractile force characteristics were measured using nerve stimulation (1.5 mA) at optimum length (*L*_*o*_), defined as the length at which the tetanic contraction force (150 Hz, 150 ms) was maximal (*F*_max⁡_). Force and length signals were sampled at 10 kHz.

*Force-velocity *(*F*-*V*)* relationships* were determined by a series of maximally stimulated concentric contractions (400 Hz) at different constant velocities (10, 20, 30, 50, 75, 100, 125, 150, 200, 250 mm/s). After stretching the muscle passively 1–1.5 mm above *L*_*o*_, the contraction started with an isometric phase during which active force reached a level which could be maintained during the shortening phase [15]. The *F*-*V* data were fitted using Hill equations and the least-squares method. Curvature (*a*/*F*_0_) and maximum shortening velocity (*V*_max⁡_) were determined. The *power-velocity curve *was obtained by multiplying, for each velocity, the velocity by its force. Maximal peak power (*P*_max⁡_) and its optimum shortening velocity (i.e., *V* at *P*_max⁡_, *V*_opt_) were determined by the fitted Hill curve. After force measurements, the GM was excised, weighed, stretched to *L*_*o*_, and rapidly frozen and stored in liquid nitrogen until further analysis. Specific force was estimated by dividing *F*_max⁡_ by the GM mass (N/g).

### 2.5. Histochemistry

As the high and low oxidative compartments of the GM are recruited task specifically [13], the fibre cross-sectional area (FCSA) in the two compartments were assessed separately. Cross-sections (10 *μ*m thick) were cut from the middle of the GM, which contained muscle fibres from both the high and low oxidative regions. The cross-sections were mounted on Vectabond (Vector Laboratories, Burlingame, CA, USA) coated slides and stored in −80°C until further analysis. 

To determine the CSA of the different fibre types in each compartment, images were captured with a 20x objective using the DMRB microscope (Leica, Wetzlar, Germany) from stained cross-sections and matched with the myofibrillar adenosinetriphosphatase (ATPase) staining to differentiate between the four fibre types (type I, type IIA, type IIX, and type IIB) [16–18]. Mean FCSA was determined from the FCSA of at least 20 fibres per type per compartment from these images which were calibrated using a slide micrometer and the set scale option in ImageJ 1.44p (National Institute of Mental Health, MD, USA), taking the pixel-to-aspect ratio into account.

### 2.6. RNA Isolation and Quantitative Polymerase Chain Reaction (qPCR)

For extraction of RNA from the high oxidative and low oxidative compartments of GM, sections were cut from the proximal and distal end of the GM, respectively. To illustrate the distribution of high and low oxidative fibres in the proximal and distal region of GM, a typical example of cross-sections stained for succinate dehydrogenase (SDH) activity is shown in Figures 3(a) and 3(b). To isolate RNA from the muscle tissue, RiboPure kit (Applied Biosystem, Foster City, USA) was used according to the manufacturer's protocol. RNA concentration and purity (260/280 nm mean ratio: 2.03; range: 1.89–2.13) were measured using spectroscopy (Nanodrop Technologies, Wilmington, DE, USA). Using the high capacity RNA-to-cDNA kit (Applied Biosystems, Foster City, USA) containing random primers in a 20 *μ*L total reaction volume, 500 ng of total RNA per muscle compartment was reverse transcribed. Tubes were heated at 25°C for 5 min, followed by 42°C for 20 min. Finally, to stop the reaction, the tubes were heated at 85°C for 5 min and stored at −80°C until used in the quantitative PCR reaction (method described previously) [19]. Expression levels of mRNA were assessed for insulin-like growth factor 1Ea (IGF-1Ea), myostatin, *α*-skeletal actin, muscle ring finger 1 (MuRF-1) and muscle atrophy F-box (MAFbx; also known as artogin 1) (see Table 1 for primer sequences). Melting curve analysis showed specific amplification and amplification efficiencies of the primers used in this study ranged from 91.3–102%. Expression levels were expressed relative to 18S rRNA using the 2^−ΔCt^ method. 18S rRNA was analysed in triplicate and the other samples in duplicate.

### 2.7. Protein Isolation and Western Blotting

For protein extraction of the high and low oxidative compartments of GM, 50 sections (20 *μ*m thick) were cut from the proximal and distal regions, respectively. Subsequently, the tissue was homogenized in ice-cold radioimmunoprecipitation assay (RIPA) buffer (Sigma-Aldrich, St. Louise MO, USA) containing protease and phosphatase inhibitor cocktail tablets (Roche, Mannheim, Germany). After centrifuging for 10 min at 12000 rpm at 4°C, the supernatant was stored at −80°C. Protein concentrations were determined using the bicinchoninic acid protein assay (Pierce, Rockford IL, USA). After denaturing the samples in SDS-PAGE sample buffer for 5 min at 90°C, 5 *μ*g of protein were subjected to SDS-PAGE and transferred to a nitrocellulose membrane (GE Healthcare, Little Chalfont, UK). Following this, the membrane was blocked with 5% ECL Advance Blocking Agent (Amersham, GE Healthcare, Little Chalfont, UK) in TBS with 0.01% Tween 20, incubated overnight at 4°C with primary antibody against phospho-S6^Ser235/236^ ribosomal protein (1 : 1000; Cell Signaling Technology) and actin (1 : 1000; Cell Signaling Technology), and followed by a specific horseradish peroxidase-conjugated polyclonal goat antirabbit secondary antibody (1 : 2000; DakoCytomation). Enhanced chemiluminescence kit (ECL Advance, Amersham, GE Healthcare, Little Chalfont, UK) was used to detect the antibody. Phospho-S6 data were normalized to actin.

### 2.8. Data Analysis

For comparison of muscle mass, maximal force, and FCSA between the different groups, these parameter values were normalized to the ratio between individual body mass and overall mean body mass at the end of the experiment. This accounted for variations in growth rate of the animals.

### 2.9. Statistical Analysis

Data was analysed using SPSS version 18.0 (SPSS Inc., Chicago, USA). When the data was normally distributed, differences between groups in maximal running velocity, GM force as function of shortening velocity, and GM power-velocity curve were tested using a mixed design repeated measures ANOVA. One-way ANOVAs were performed to assess the differences in maximal force, FCSA, and gene and protein expression levels between the three groups. Bonferroni post hoc analysis was performed when a significant main effect of group was found. When data was not normally distributed according to the Shapiro-Wilk test (IGF-1Ea, MuRF-1 and MAFbx mRNA, and phospho-S6 levels in both compartments), Kruskal-Wallis Test was performed to test for differences between the three groups. In case of significant main effects, Mann-Whitney *U* Test was used to compare the different groups. The *P* value was adjusted using the Bonferroni method. The results are presented in mean ± SE. Significance was considered at *P* < 0.05.

## 3. Results and Discussion

In total, 20 rats were trained. However, three rats of the PT group did not complete enough training sessions successfully and were therefore excluded. During force measurements, GM of three rats (two rats of the PET and one of the control group) were damaged due to technical problems, resulting in 3 additional exclusions. Therefore, the ultimate number of rats used for all analyses were for control *n* = 9, for PT *n* = 7, and for PET *n* = 8. The initial and final body masses were not significantly different between the three groups (Table 2).

### 3.1. Effects of Training on Functional Performance, Contractile Force Characteristics and Hypertrophy of Rat GM

**Figure 1:**
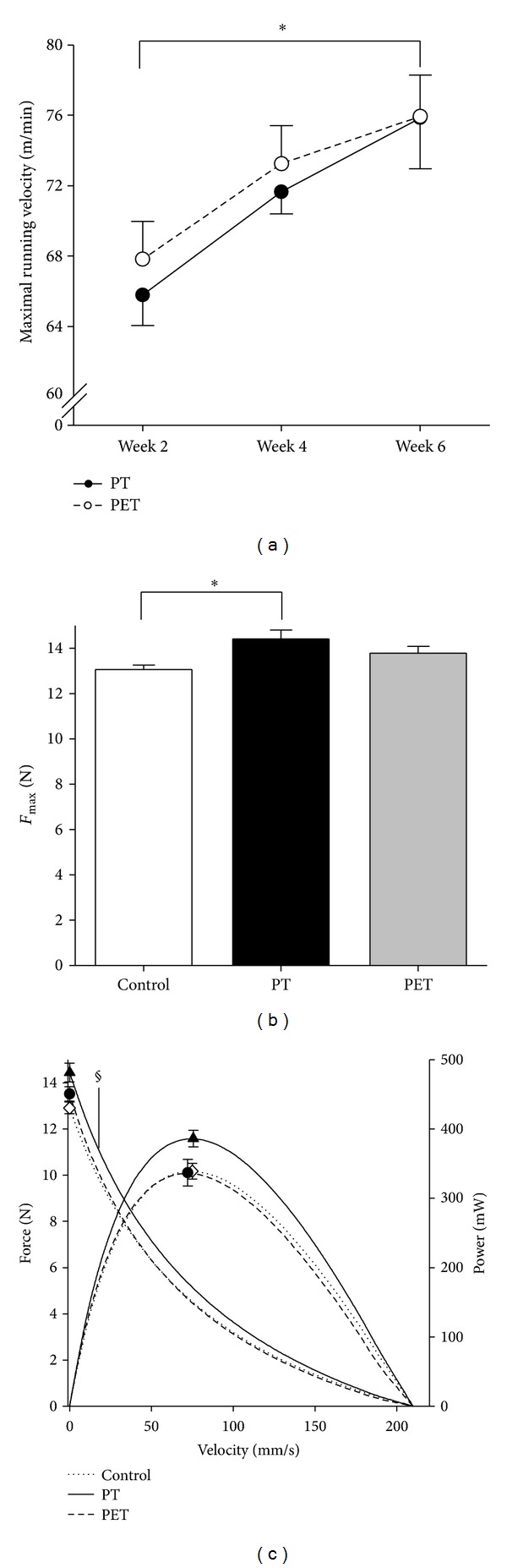
Effects of training on running performance and GM force characteristics of the rats. Effects of peak power training alone (PT) and peak power training in combination with endurance training (PET) on (a) maximum running velocity, (b) maximal force (*F*_max⁡_), and (c) force-velocity and power-velocity curves of the rat medial gastrocnemius muscle. Forces as a function of contraction velocity were significantly higher after PT compared to those of controls. Symbols (control: white diamond, PT: black triangle, PET: black circle) on the force velocity curve indicate the isometric *F*_max⁡_ and symbols on the power curve indicate maximal peak power at optimum shortening velocity. All values are mean ± SE. *= significant difference between the groups (*P* < 0.05); ^§^= significantly different from control (*P* < 0.05).

The maximal running velocity increased 13.5% (*P* = 0.004) throughout the last 4 weeks of training in both groups (Figure 1(a)), and was not significantly different between the PT and PET group. After PT, *F*_max⁡_ was 10% higher compared to that of controls (*P* = 0.015), whereas after PET, mean *F*_max⁡_ was neither significantly different from controls nor PT, indicating that the additional endurance training attenuated the increase in *F*_max⁡_ (Figure 1(b)). For the PT group, also forces as a function of velocity were significantly higher (*P* = 0.040) than those of controls (Figure 1(c)). Similar to the results for *F*_max⁡_, for the PET group, forces as a function of velocity did neither differ from those of controls nor from those of PT group. The power-velocity curves were not significantly different between the three groups (*P* = 0.111). A tendency (*P* = 0.071) was found towards a difference in *P*_max⁡_ between the three groups, whereby *P*_max⁡_ of the PT group was 13.9% higher (386.1 ± 11.9 mWatt) than that of controls (339.1 ± 11.4 mWatt) and 14.7% higher (336.6 ± 19.1 mWatt) compared to that of the PET group. While *F*_max⁡_ of the PET group could not be shown to be higher after training, maximal running velocity increased throughout the training. The increase in running performance may be caused by improved recruitment and coordination of agonists and antagonists as shown after resistance training [20–23], or *F*_max⁡_ of synergistic muscles may have increased after training, which may also have contributed to the better running performance.

The higher *F*_max⁡_ after PT (isometrically as well as as a function of velocity) may be explained by either changes in cross-bridge kinetics and/or Ca^2+^-sensitivity, hypertrophy, or increased specific force. Since no significant differences between groups were observed in *V*_max⁡_ and *a*/*F*_0_, representing the force-velocity curvature, training was unlikely to induce changes in the number of sarcomeres in series, cross-bridge kinetics, and/or Ca^2+^-sensitivity.

**Figure 2:**
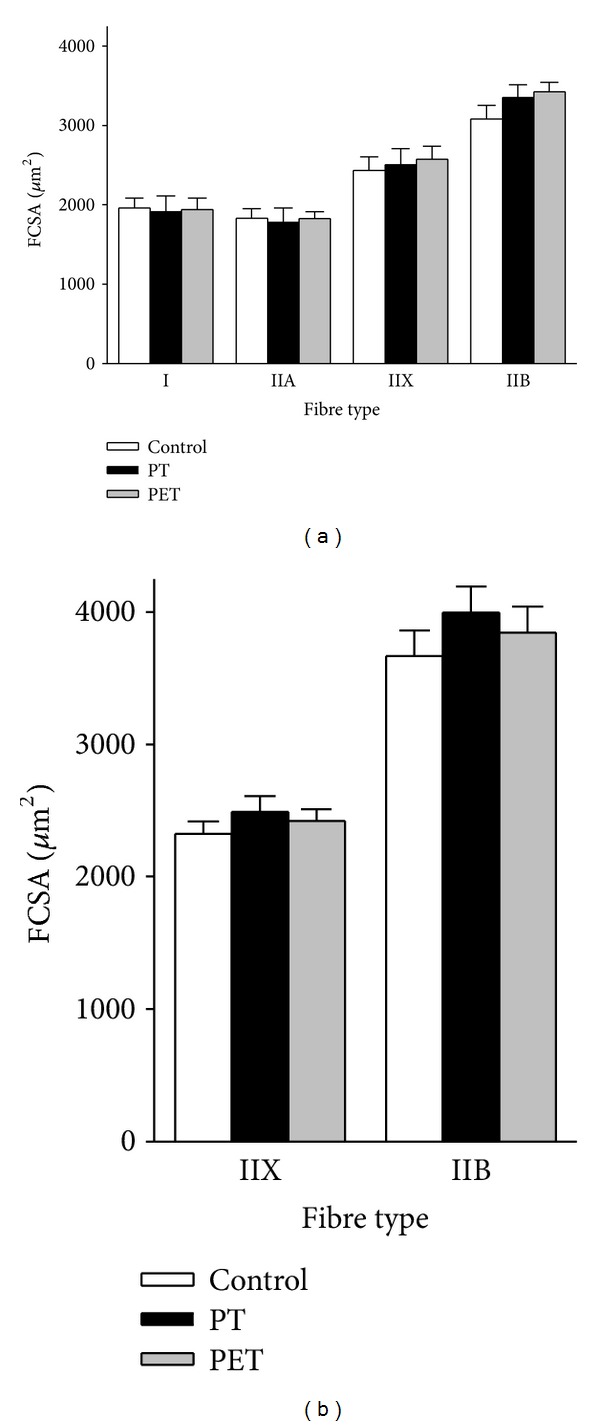
Effects of training on muscle fibre size. Peak power training alone (PT) and peak power training in combination with endurance training (PET) did not change the fibre cross-sectional area (FCSA) in the high oxidative (a) and low oxidative (b) compartments of the rat medial gastrocnemius muscle. All values are mean ± SE. *= significant difference between the groups (*P* < 0.05).

As the GM is a compartmentalized muscle, of which the high oxidative compartment of controls consisted of 23.7 ± 1.0% type I, 22.0 ± 1.7% type IIA, 34.8 ± 2.2% type IIX, and 19.5 ± 1.2% type IIB fibres and the low oxidative compartment for 17.8 ± 3.1% of type IIX and 82.2 ± 3.1% of type IIB fibres (data not shown), we investigated whether fibre type-specific changes in CSA may explain the attenuated increase in *F*_max⁡_ after PET. In both compartments, training did not affect the FCSA of any fibre type (Figure 2). However, the tendency (*P* = 0.065) observed towards an 8% higher GM mass after PT compared to controls (Table 2) and unchanged specific force after training (control 18.95 ± 0.46 N/g; PT 19.32 ± 0.66 N/g; PET 19.21 ± 0.29 N/g) suggests that the increase in *F*_max⁡_ seen after PT may be due to muscle hypertrophy.

### 3.2. Effects of Training on Genes Involved in the Regulation of Muscle Hypertrophy and Atrophy of GM

To obtain an indication of the regulatory mechanisms underlying the training-induced adaptations and the possible interaction, we investigated steady-state mRNA expression levels of two key growth factors, insulin-like growth factor- (IGF-) 1Ea and myostatin, and their downstream targets involved in the regulation of muscle size and maximal force. IGF-1 may increase the rate of mRNA translation by activating the phosphoinositide 3-kinase (PI3 K)/Akt/mammalian target of rapamycin (mTOR) signalling pathway and reduce the rate of protein degradation via its inhibitory effect on the expression of the muscle specific E3 ubiquitin ligases, muscle ring finger- (MuRF-) 1, and muscle atrophy F-box (MAFbx) [24]. In contrast, myostatin has opposite effects and is considered a negative regulator of muscle growth [25–27] by reducing Akt/mTOR signalling [28, 29] and stimulating expression of MAFbx and MuRF-1 [27]. To assess the net effects of changes in IGF-1 and myostatin expression on Akt/mTOR signalling and protein degradation, we quantified phospho-S6 protein levels and mRNA levels of *α*-skeletal actin, MuRF-1 and MAFbx, respectively.

*Total RNA and 18S RNA.* In controls, total RNA content and 18S rRNA (2^Ct^) per ng total RNA were not different between the high and low oxidative compartments. In both compartments, training did not affect these parameters. Therefore, mRNA expression levels of the different target genes were expressed relative to 18S and considered as estimates of their mRNA concentrations.

**Figure 3:**
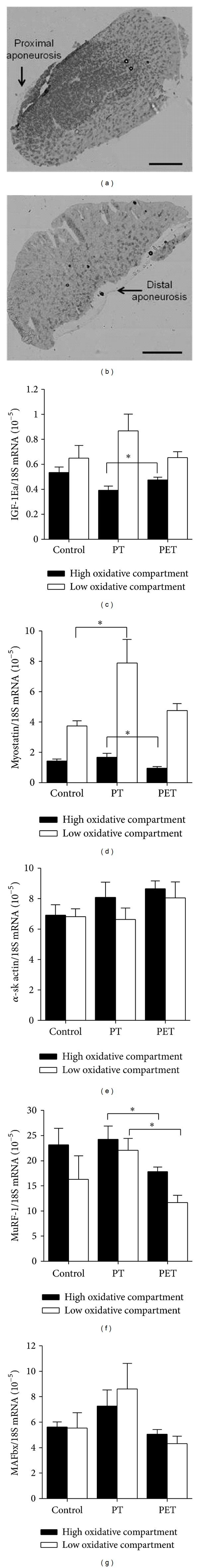
Effects of training on gene expression of regulatory factors of muscle protein synthesis and degradation and downstream targets. (a) and (b) show typical examples of cross-sections from the proximal (i.e., high oxidative compartment) and distal regions (i.e., low oxidative compartment), respectively, of rat medial gastrocnemius muscle, which were stained for succinate dehydrogenase activity. These images demonstrate that the proximal region predominantly consists of high oxidative fibres whereas the distal region mainly consists of low oxidative fibres. RNA was extracted from these regions. Effects of peak power training alone (PT) and peak power training in combination with endurance training (PET) are shown on the gene expression of IGF-1Ea (c), myostatin (d), *α*-skeletal actin (e), MuRF-1 (f), and MAFbx (g) in the high (black bars) and low (white bars) oxidative compartments of GM. mRNA expression is relative to 18S rRNA. All values are mean ± SE. Bars indicate 1000 *μ*m. *= significant difference between the groups (*P* < 0.05). IGF-1Ea: insulin-like growth factor 1Ea, MAFbx: muscle atrophy F-box, MuRF-1: muscle ring finger 1.

**Figure 4:**
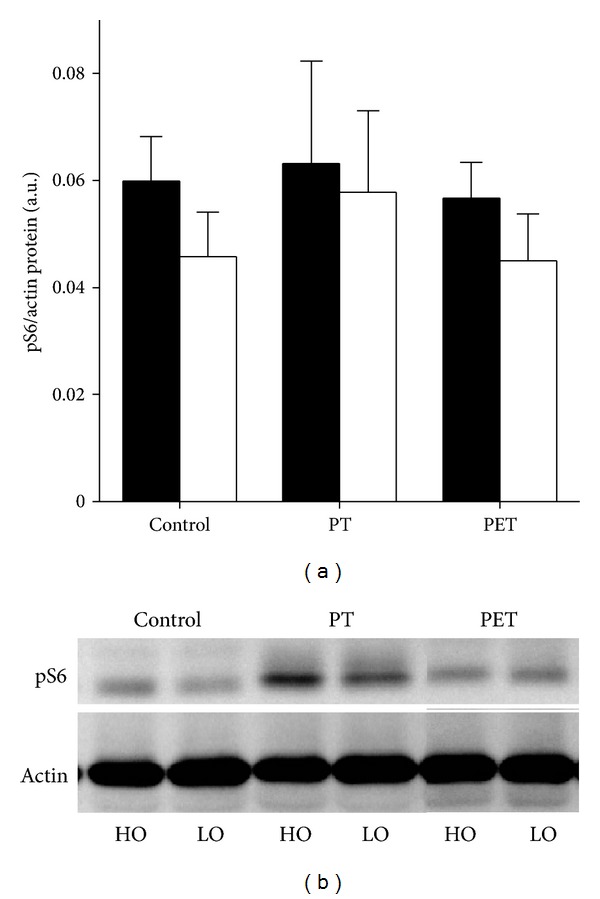
Effects of training on the phospho-S6 levels. Peak power training alone (PT) and peak power training in combination with endurance training (PET) did not affect phospho-S6 protein content in the high (black bars) and low (white bars) oxidative compartments of rat medial gastrocnemius muscle. Protein content was normalized to actin levels. All values are mean ± SE.

*Effects of Training on High Oxidative Compartment.* After 6 weeks of training, PT resulted in lower IGF-1Ea mRNA levels (Figure 3(c); *P* = 0.045) and higher myostatin and MuRF-1 mRNA levels compared to PET (Figures 3(d) and 3(f); *P* = 0.025 and *P* = 0.045, resp.). No significant differences were observed in mRNA levels of *α*-skeletal actin and MAFbx and protein levels of phospho-S6 between the three groups (Figures 3(e), 3(g), and 4). Thus, additional endurance training on top of peak power training prevented the reduction of the IGF-1Ea mRNA expression and increase of myostatin mRNA and therefore prevented an increased expression of the E3 ubiquitin ligase MuRF-1. Since PT increased *F*_max⁡_, an anabolic status of the muscle was expected with higher IGF-1Ea and lower myostatin mRNA levels [30, 31]. Although IGF-1 is known for its role in muscle growth, recently its role in loading-induced muscle hypertrophy has been questioned [32–34]. Functional overloading in mice was shown to induce hypertrophy without a functional IGF-1 receptor [34] and acute resistance exercise in mice did not change IGF-1 receptor phosphorylation [32], indicating that hypertrophy can occur in an IGF-1 independent manner.

*Effects of Training on Low Oxidative Compartment.* Also in the low oxidative compartment, myostatin and MuRF-1 mRNA levels were significantly different after training (*P* = 0.008 and *P* = 0.010, resp.), whereas IGF-1Ea, *α*-skeletal actin, and MAFbx remained unchanged (Figure 3). PT resulted in a 2.1-fold higher myostatin mRNA content compared to that in controls (*P* = 0.008), while myostatin mRNA content after PET was unchanged. PT also induced a 1.9-fold higher MuRF-1 mRNA expression compared to that after PET (*P* = 0.012). Similar to the high oxidative compartment, additional endurance training, performed by PET, inhibited the substantial increase in myostatin mRNA content and induced a decrease in MuRF-1 mRNA content compared to PT. Since phospho-S6 protein levels remained unchanged after training, the higher myostatin levels after PT were unlikely to inhibit Akt/mTOR signalling but rather seem to have increased the rate of protein degradation which is reflected by higher MuRF-1 mRNA levels. Note, that as we assessed mRNA levels of the E3 ubiquitin ligases, it remains uncertain whether their protein levels were also increased after PT. Although the elevated catabolic status within the muscle of the PT group was not accompanied by a decrease in FCSA, and PT even resulted in a 10% higher *F*_max⁡_, the substantial increase in myostatin mRNA after PT in the low oxidative compartment was unexpected. However, in agreement with our observations, elevated myostatin mRNA, protein and serum levels were observed after 3, 6, and 12 weeks of resistance training in humans which was also accompanied with an increase in maximal force [35, 36]. In addition, high myostatin levels may not always be associated with smaller FCSA. Paradoxically, myostatin is more abundantly expressed in fast muscles (i.e., m. Extensor digitorum longus) consisting predominantly of large type IIX and IIB fibres compared to slow muscles (i.e., m. Soleus) [11, 37]. Furthermore, the percentage type IIB fibres of a muscle were shown to be positively related (*r* = 0.725) to its myostatin mRNA content [38], suggesting that although myostatin may be a negative regulator of muscle fibre size in general, the magnitude of its impact seems fibre type specific.

## 4. Conclusions

We investigated whether by using task-specific recruitment of GM, additional endurance training would not interfere with the peak power training induced adaptive responses. While the increase in maximal running velocity was similar in both training groups, *F*_max⁡_ of GM was only increased after peak power training alone and not after concurrent peak power and endurance training. In addition, in both, the high and low oxidative compartments, additional endurance training performed by PET induced different expression levels of genes involved in protein synthesis and degradation than PT, suggesting an interaction between the training response of peak power and endurance training. Thus, even by using task-specific recruitment of the compartmentalized rat GM, additional endurance training interfered with the adaptive response of peak power training and attenuated the increase in *F*_max⁡_ after peak power training. Further research is needed to investigate how these two training types can best be combined to prevent interference at the cellular level and improve the size and maximal force of the entire muscle.

## Figures and Tables

**Table 1:** Sequences of the specific primers used in the quantitative PCR analyses.

Target mRNA	PCR primer sequence 5′–3′	
	Forward	Reverse
18S rRNA	CGAACGTCTGCCCTATCAACTT	ACCCGTGGTCACCATGGTA
IGF-1Ea	AAGCCTACAAAGTCAGCTCG	TCAAGTGTACTTCCTTCTGAGTC
myostatin	GGTCCCGGAGAGACTTTGG	CGACAGCACCGCGATTC
*α*-skeletal actin	CGACATCGACATCAGGAAGGA	GGTAGTGCCCCCTGACATGA
MuRF-1	TGCCCCCTTACAAAGCATCTT	CAGCATGGAGATGCAATTGC
MAFbx	TGAAGACCGGCTACTGTGGAA	CGGATCTGCCGCTCTGA

PCR: polymerase chain reaction, IGF-1Ea: insulin-like growth factor 1Ea, MuRF-1: muscle ring finger 1, MAFbx: muscle atrophy F-box.

**Table 2:** Initial and final body and medial gastrocnemius muscle (GM) mass.

Group	Body mass (g) ± SE		Muscle mass (g) ± SE
	Initial body mass	Final body mass	Final GM mass
Control (*n* = 9)	208.1 (±4.1)	255.2 (±7.4)	0.691 (±0.013)
PT (*n* = 7)	201.7 (±3.5)	250.1 (±3.7)	0.748 (±0.021)
PET (*n* = 8)	211.0 (±3.6)	254.5 (±2.3)	0.717 (±0.014)

PT: peak power training; PET: combined peak power training and additional endurance training. Values are mean ± SE.
